# Supplementary material for: Spray Flame Synthesis and Multiscale Characterization of Carbon Black–Silica Hetero-Aggregates
Source: Nanomaterials (Basel). 2023 Jun 20;13(12):1893. doi: 10.3390/nano13121893 (PMC10302686; doi:10.3390/nano13121893)
Supplement: Supplementary file 1 [file nanomaterials-13-01893-s001.zip › nanomaterials-2462688-supplementary.pdf]

nanomaterials

Supplementary Materials

Spray Flame Synthesis and Multiscale Characterization of Carbon Black–Silica Hetero-Aggregates

Simon Buchheiser *, Ferdinand Kistner, Frank Rhein and Hermann Nirschl *

Process Machines, Institute of Mechanical Process Engineering and Mechanics, Karlsruhe Institute of Technology, 76131 Karlsruhe, Germany
* Correspondence: simon.buchheiser@kit.edu (S.B.); hermann.nirschl@kit.edu (H.N.)

Table S1. Experimental conditions of the stoeber process

Name	NH_3 / ml	H_2O / ml	EtOH / ml	TEOS / ml	Temperature / °C
60 nm	7.2	11.8	230	24	50
40 nm	6.1	10	230	22.5	50
30 nm	6.1	19.5	230	11.9	60
10 nm	7.2	4.5	230	24	50

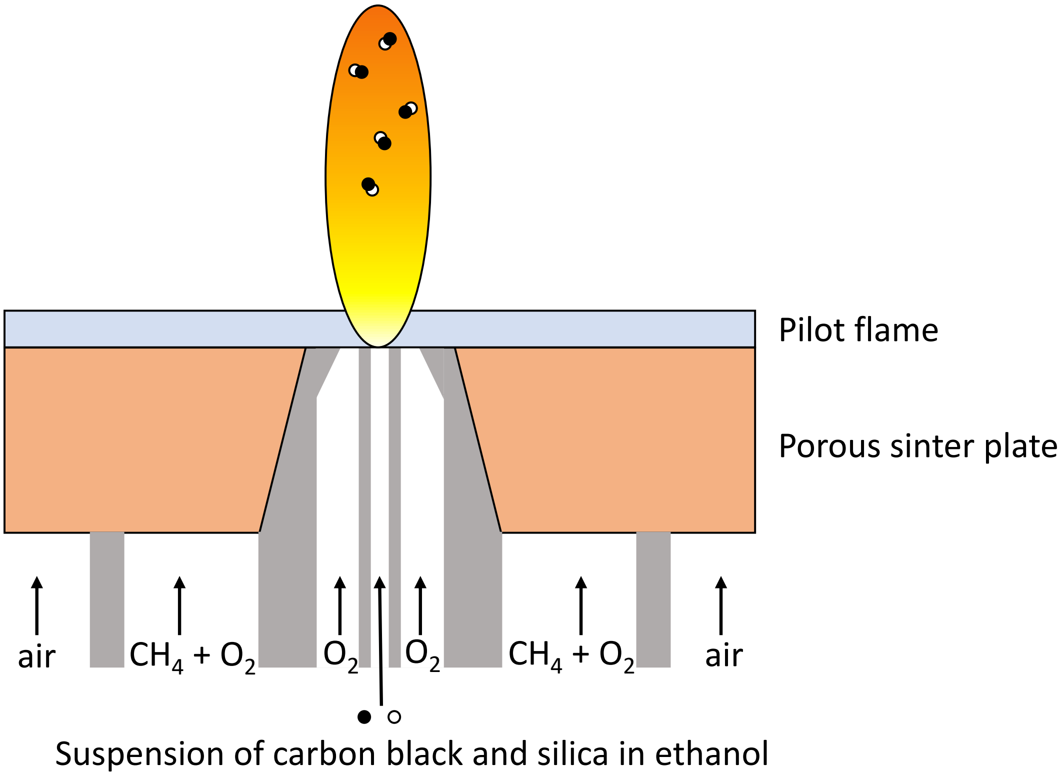

Figure S1. Schematic drawing of the used SpraySyn burner. Not to scale.

Table S2. Flame parameters of the SpraySyn burner

**CH_4 pilot /slm**	**O_2 pilot /slm**	**O_2 dispersion gas /slm**	**Air sheath gas / slm**	**Volume flow suspension /$ml \cdot min^{-1}$**	**Concentration carbon black in suspension / mass-%**	**Ratio silica to carbon black / $g \cdot g^{-1}$**
2	12	10	120	3	0.5	0.2-5
